# RbTiOPO_4_ cascaded Raman operation with multiple Raman frequency shifts derived by Q-switched Nd:YAlO_3_ laser

**DOI:** 10.1038/srep33852

**Published:** 2016-09-26

**Authors:** Yanmin Duan, Haiyong Zhu, Yaoju Zhang, Ge Zhang, Jian Zhang, Dingyuan Tang, A. A. Kaminskii

**Affiliations:** 1 College of Physics and Electronic Information Engineering, Wenzhou University, Wenzhou 325035, China; 2 Fujian Institute of Research on the Structure of Matter, Chinese Academy of Sciences, Fuzhou 350002, China; 3 School of Physics and Electronic Engineering, Jiangsu Normal University, Xuzhou 221116, China; 4 Institute of Crystallography, Russian Academy of Sciences, Moscow 119333, Russia

## Abstract

An intra-cavity RbTiOPO_4_ (RTP) cascade Raman laser was demonstrated for efficient multi-order Stokes emission. An acousto-optic Q-switched Nd:YAlO_3_ laser at 1.08 μm was used as the pump source and a 20-mm-long *x*-cut RTP crystal was used as the Raman medium to meet the X(Z,Z)X Raman configuration. Multi-order Stokes with multiple Raman shifts (~271, ~559 and ~687 cm^−1^) were achieved in the output. Under an incident pump power of 9.5 W, a total average output power of 580 mW with a pulse repetition frequency of 10 kHz was obtained. The optical conversion efficiency is 6.1%. The results show that the RTP crystal can enrich laser spectral lines and generate high order Stokes light.

Stimulated Raman scattering (SRS) is an efficient non-linear frequency conversion process to enrich laser spectral lines. Especially for intra-cavity crystalline Raman laser pumped by all-solid-state laser^1^, it has the advantages of low threshold and high overall efficiency by making full use of high power density inside the laser cavity, as well as compact and rugged configuration. Recently, many kinds of crystals have been discovered with high Raman gain and adopted for efficient Raman laser generation, such as YVO_4_^2^,^3^, GdVO_4_^4^, SrWO_4_^5^,^6^, KGd(WO_4_)_2_^7^ and diamond^8^,^9^.

RbTiOPO_4_ (RTP) and its isomorphs KTA and KTP are superior non-linear optical materials. They have been widely used to generate eye-safe lasers based on optical parametric oscillation^10^,^11^,^12^,^13^,^14^. Beside the attractive application in parametric conversions, RTP with high optical damage threshold is also a great Raman crystal candidate for high power Stokes output^15^,^16^,^17^,^18^. In resent years, the RTP and its isomorphs also have been used for THz radiation via stimulated polarition scattering^19^,^20^. Compared with the other Raman crystals, the RTP and its isomorphs own smaller Raman shift with strong Raman gain coefficient. They permit generating closely spaced lines with multi-order Stokes radiation through the cascade stimulated Raman scattering. Up to now, KTP and KTA have been reported for efficient Raman generation^21^,^22^,^23^,^24^,^25^. In 2008, KTP crystal Raman output 1.02 W simultaneous multi-order Stokes radiation with Raman shift of 271 cm^−1^ was reported by Chang *et al*. for the first time^21^. Next year, the first and second Stokes of KTA crystal Raman laser was also reported by Liu *et al*.^22^,^23^. The second Stokes at 1120 nm with the average output power of 0.63 W and conversion efficiency of 9.4% was generated in diode-end-pumped Nd:YAG/KTA Raman configuration. Our group also reported multi-order Stokes radiation in a KTA crystal with maximum output power of 1.12 W^26^. The RTP has strong Raman emission at frequency shifts of 271 and 687 cm^−1^ which are slightly different comparing to KTA and KTP. In 2006, Pearce *et al*.^27^ reported the first Stokes of RTP emitting at 1096 nm with the frequency shift around 271 cm^−1^. Both 1064 nm and 1096 nm outputted with an average power of about 200 mW at each wavelength were achieved under the pump power of about 3.5 W. In this paper, the intra-cavity RTP cascade Raman operation with multiple frequency shifts was investigated and an acousto-optic Q-switched Nd:YAlO_3_ laser was assigned as the pump source. Multi-order Stokes emission around 1.1–1.2 μm was realized based on cascaded and cross-cascaded Raman conversion with frequency shifts of ~271, ~559 and ~687 cm^−1^.

As reported by Kugel *et al*.^28^ and Watson^17^, the strongest Raman scattering of RTP with the orthorhombic non-centro symmetric space group *Pna*21 and point group *C*_2*v*_ (*mm*2) was obtained from the *A*1 (*ZZ*) geometry. An RTP single crystal 4* × *4* × *20 mm^3^ in size, along x-axis cut was used as the Raman crystal. A b-cut, 0.9 at.% doped Nd:YAlO_3_ crystal with a size of Φ3.6 × 7 mm^3^ was used as the laser crystal for polarized laser oscillation. The polarization of the fundamental light is fixed parallel to the z-axis of RTP to meet the X(Z,Z)X Raman configuration in this experiment. A schematic diagram of the experimental setup is shown in Fig. 1.

The laser crystal was end-pumped by an 808 nm fiber coupled laser diode array with a re-imaged pumping spot size of about 320 μm in diameter. An acousto-optic Q-switch module (AOM, Gooch & Housego Co.) with 30-mm in length was inserted between the Nd:YAlO_3_ and RTP crystals for Q-switching operation. The fundamental and Raman oscillation shared the same cavity that has a total length of 70 mm and is comprised of an input mirror (IM) and the output coupler (OC). IM was high-transmission (HT, T > 95%) coated for the pump light at 808 nm and high reflection (HR, R > 99.9%) coated for the fundamental and Stokes lights from 1.0 to 1.2 μm. In order to realize the multi-Stokes, the output coupler OC with partial reflection among 1.1–1.2 μm was coated. The measured transmittance of the OC for the wavelengths relevant to laser system is shown in Fig. 2 and Table 1.

The Raman threshold decreased with the pulse repetition frequency. The output power of the leaked fundamental light at 1.08 μm from the OC was measured after filtering by a mirror with partial-reflection coated at 1.08 μm and high-reflection coated above 1.11 μm. It shows that the leaked output power of the fundamental light increased with the pulse repetition frequency. At the pulse repetition frequency of 5 kHz, the leaked power of the fundamental light was about 24 mW, which increased to about 55 mW at the frequency of 15 kHz. The leaked output power of fundamental light had been deducted from the total output power. The average output power of the Raman output versus the incident diode pump powers at the pulse repetition frequency of 5, 10, and 15 kHz are shown in Fig. 3. Under an incident pump power of 9.5 W, the maximum total output power was 580 mW at a pulse repetition frequency of 10 kHz, corresponding to a conversion efficiency of 6.1%.

The output laser spectra without filtering were measured by the grating monochromatar (model Omni-λ500 with the slit of 0.05 mm and the resolution of 0.05 nm). The number of Stokes lines increased by enlarging of pump power and reducing of pulse repetition frequency. Multi-wavelengths lines with the wavelength among the region from 1.08 to 1.22 μm were detected with an incident diode pump power of 9.5 W and a pulse repetition frequency of 10 kHz. The measured spectra of the laser output was displayed in Fig. 4. Accompany with the output of multi-Stokes, the yellow light irradiated from the RTP crystal was detected, which was converted by frequency mixing between multi-Stokes lights. The spectra detected by AvaSpec-3648 Fiber Optic Spectrometer was shown in Fig. 5. The phase match angle of the x-cut RTP crystal which is close to type-II phase match angle (θ = 87°, ϕ = 0°) for the frequency doubling of second-stokes light at 1147 nm, resulted in the strongest line of 573.5 nm. The other weaker lines were sum-frequency between the fundamental and Stokes lines. Except for the line of 546 nm, the lines in Fig. 5 can be calculated by frequency mixing among the lines in Fig. 4. According to our laser system, the 546 nm could be converted by frequency mixing of fundamental light and first Stokes light at 1105 nm with weak vibration mode of 213 cm^−1^. The yellow light was weak since all the polarization of the fundamental and Stokes lines is in the cavity were parallel to the Z axis of RTP and can’t meet the type-II phase match for frequency mixing. In Fig. 4, the line of 1105 nm was not detected due to low power level and HR coated of output coupler. The wavelengths of the laser oscillating in the cavity are listed in Table 1. It can be easily come to conclusion that the lines 1112, 1147, 1184 and 1123 nm are the first to fourth order Stokes converted by cascading Raman conversion with the strongest frequency shift of 271 cm^−1^. The 1149 nm and 1166 nm are the first Stokes lines with the Raman shifts of 559 and 687 cm^−1^, respectively. Other stokes lines were converted by cross-cascading Raman conversion with mixed frequency shifts as listed in Table 1. The above four vibration modes with the frequencies of 213, 271, 559 and 687 cm^−1^ can be found in spontaneous Raman spectra of RTP^17^,^18^. In the Table 1, we also listed the predicted Stokes wavelengths converted by above four vibration modes and measured center wavelengths for comparison.

Comparing with reports on similar multi-Stokes output from KTP^21^ and KTA^26^, the RTP resulted in more complex spectral. The results show that the RTP crystal can enrich laser spectral lines and generate high order Stokes light. The oscillation of multi-Stokes also leaded to low power stability. The power stability of the Raman output was investigated with a power meter. It was found that the power fluctuation reached 10% at the maximum output power. The temporal pulse profiles of laser output were recorded by an InGaAs free-space photo detector, and displayed on a 500 MHz oscilloscope (Model DPO3052B). Because the multi-Stokes light was close to each other in the spectra, we can’t separate each Stokes for pulse profile measurement. We only recorded the average pulse profiles of the multi-wavelength Stokes light and the fundamental light. Fig. 6 shows the temporal pulse profiles for Stokes light and fundamental light at the pulse repetition frequency of 10 kHz and an incident pump power of 9.5 W. The pulse widths of the Stokes light and fundamental light were about 8.4 ns and 14.2 ns. The higher order Stokes always leads to lower conversion efficiency for larger loss between Stokes photon and fundamental photon. Because only one output coupler was available in our experiment, conversion efficiency and output power might be improved based on further optimizing of the output coupler.

In conclusion, we demonstrated the intra-cavity RTP cascade Raman operation derived by a LD end-pumped acousto-optic Q-switched Nd:YAlO_3_ laser at 1.08 μm. A 20-mm-long *x*-cut RTP crystal was used as the Raman medium to meet the X(Z,Z)X Raman configuration. Efficient multi-order Stokes light emission was realized, which contains Stokes lines converted with Raman frequency shifts of 271, 559 and 687 cm^−1^. At an incident pump power of 9.5 W and a pulse repetition frequency of 10 kHz, the total average output power of the multi-Stokes light was 580 mW and the diode to multi-Stokes light conversion efficiency was about 6.1%. RTP resulted in more complicated spectra due to multiple vibration modes participated.

## Additional Information

**How to cite this article**: Duan, Y. *et al*. RbTiOPO_4_ cascaded Raman operation with multiple Raman frequency shifts derived by Q-switched Nd:YAlO_3_ laser. *Sci. Rep.* **6**, 33852; doi: 10.1038/srep33852 (2016).

## Figures and Tables

**Figure 1:**
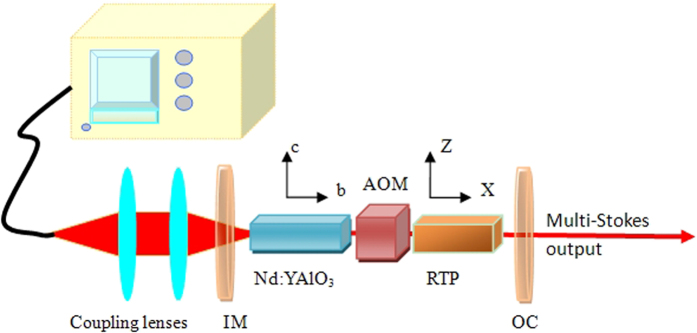
Experimental configuration of the RTP intracavity Raman laser pumped by Nd:YAlO_3_ laser.

**Figure 2:**
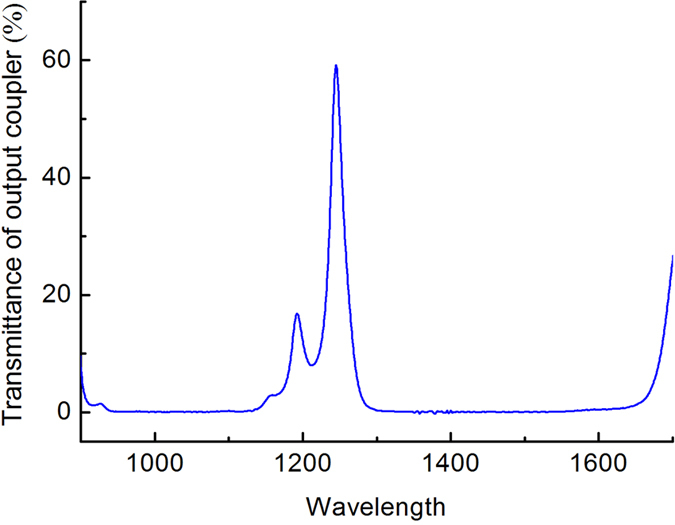
Measured transmittance of the output coupler.

**Figure 3:**
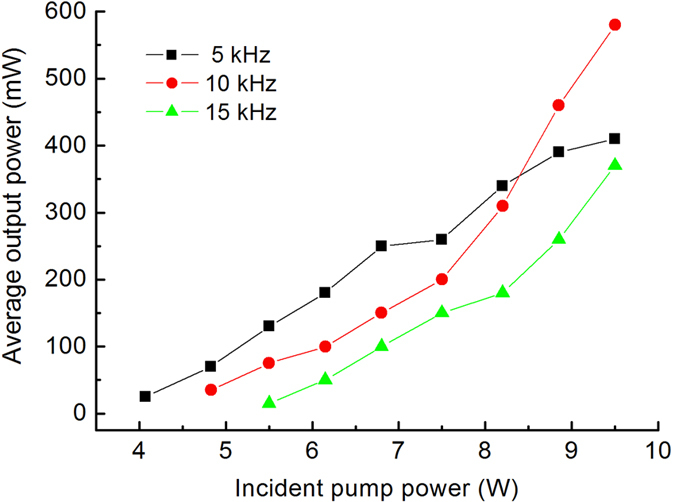
Average output power of the Raman laser versus incident pump power under different pulse repetition frequencies.

**Figure 4:**
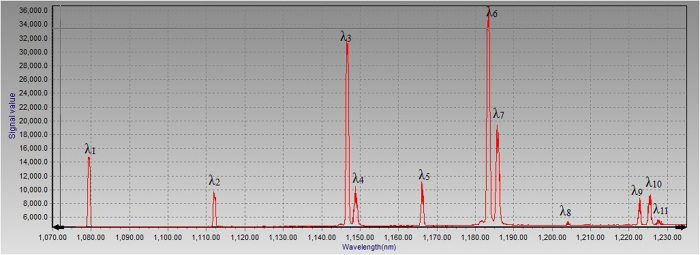
Measured spectra of Raman laser with multi-stokes output.

**Figure 5:**
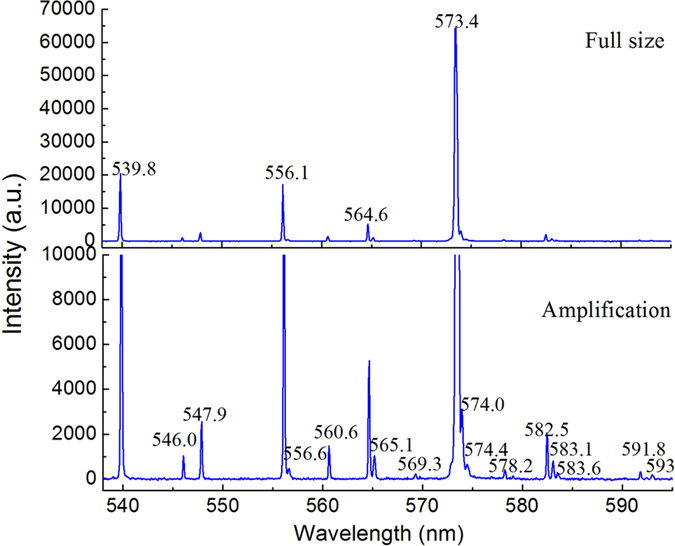
Spectrum of yellow light irradiated from the RTP crystal.

**Figure 6:**
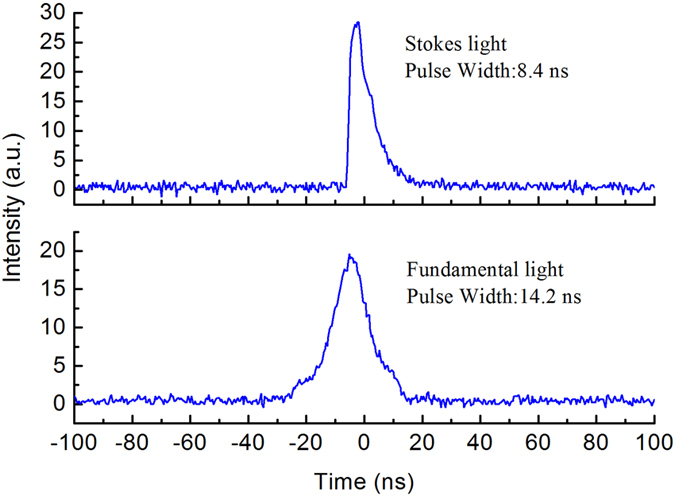
Temporal pulse profile for Stokes light and fundamental light.

**Table 1:** Transmittances of OC and potential conversion for the Wavelengths relative to laser system.

Line in Fig. 4	Predicted Wavelength (nm)	Transmittance (%)	Contributed frequency (cm^−1^) ω_R1_ = 213, ω_R2_ = 271, ω_R3_ = 559, ω_R4_ = 687	Measured center wavelength (nm)
λ_1_	1079.6	< 0.1	0	1079.6
/	1105.0	< 0.1	ω_R1_	1104.7
λ_2_	1112.1	< 0.1	ω_R2_	1112.2
λ_3_	1146.7	1.5	2ω_R2_	1146.8
λ_4_	1148.9	1.8	ω_R3_	1148.8
λ_5_	1166.1	3.1	ω_R4_	1166.2
λ_6_	1183.5	9.2	3ω_R2_	1183.6
λ_7_	1185.9	11.8	ω_R2_ + ω_R3_	1185.9
λ_8_	1204.1	9.5	ω_R2_ + ω_R4_	1204.2
λ_9_	1222.7	10.5	4ω_R2_	1222.9
λ_10_	1225.2	12.2	2ω_R2_ + ω_R3_	1225.4
λ_11_	1227.8	14.6	2ω_R3_	1227.9
